# Quantifying the Sources of the Severe Haze over the Southern Hebei Using the CMAQ Model

**DOI:** 10.1155/2013/812469

**Published:** 2013-09-15

**Authors:** Jing Yang, Pu Zhang, Chenchen Meng, Jie Su, Zhe Wei, Fenfen Zhang, Wei Wei, Xiujuan Zhao

**Affiliations:** ^1^Department of Environmental Engineering, Hebei University of Engineering, Handan, Hebei 056038, China; ^2^State Environmental Protection Key Laboratory of Sources and Control of Air Pollution Complex, Beijing 100084, China

## Abstract

The Southern Hebei of China has experienced an obvious increase of the haze occurrence frequency in the recent years. It has turned out to be one of the most seriously polluted areas in China. This study is aimed at quantifying the sources of the serious haze pollution over the Southern Hebei area, using the Mesoscale Modeling System Generation 5 (MM5) and the Models-3/Community Multiscale Air Quality Model (CMAQ) modeling system. The sectoral contributions by the local and the surrounding regions to the fine particulate matter (PM_2.5_) concentrations in the two representative cities, Shijiazhuang and Xingtai, were analyzed by applying the method of scenario analysis. It will provide useful information to the policy making in the severe air pollution control in the Southern Hebei area.

## 1. Introduction

In recent years, regional haze has been one of the most disastrous weather events in China [1]. Along with the economic development, many developed regions in China such as the Beijing-Tianjin-Hebei (BTH) region, Pearl River Delta, Sichuan Basin, and Yangtze River Delta have suffered serious haze pollution [2]. Hebei province is located in northern China. Its occurrence frequency of haze has experienced significant increase in recent years [3], especially in its central and southern parts. The average Air Quality Index (AQI) in the first quarter of 2013 released by the Ministry of Environmental Protection (MEP) showed that the top five most polluted cities were all in Hebei province, including Shijiazhuang, Xingtai, Baoding, Handan, and Tangshan. MEP also reported that the large scope and long term of haze pollution in January and February 2013 was the main reason of highest AQI in these cities. In particular in January 2013, Hebei province suffered haze pollution during 8–17, 22–24, and 27–30, 17 days in total. The intensity, scope, and durability were rare in history. 

It is necessary and urgent to carry out haze research in this region. The effective control of haze pollution should be based on the scientific understanding of its source and formation mechanism. Zhao et al. [4] and Wang et al. [5] have conducted regional scale modeling over Hebei and the surrounding areas and estimated the contributions of anthropogenic emission in the local and surrounding regions to PM_2.5_ in Shijiazhuang and Xingtai city. They concluded that the major contributors were the Hebei area (72.6%), Shanxi (10.4%), Henan (3.7%), and Shandong (3.6%) to Shijiazhuang City. 

This study further quantified the sectoral contributions from the major emission sectors to the PM_2.5_ concentrations in Shijiazhuang and Xingtai city in July and December, 2007. The scenario analysis method was applied. This study is aimed at providing scientific information for the future air pollution control and improvement in the Southern Hebei area. 

## 2. Methodology

### 2.1. Modeling Domain and Period

**Figure 1:**
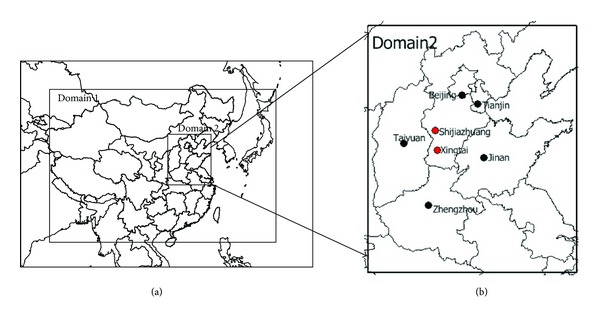
(a) Two domains used in CMAQ modeling. Domain 1: 36 km over most of East Asia with 164 × 97 grids; domain 2: 12 km over most of eastern China with 93 × 111 grids. (b) Location of target cities in domain 2 and the range of domain 2.

Lambert projection with the two latitudes of 25°N and 40°N was adopted in the whole simulation. The domain origin was 34°N, 110°E. Two nested domains were chosen in this study considering that grid spatial resolution had a significant impact on the accuracy of simulation, as shown in Figure 1(b). The inner domain included Hebei and five surrounding provinces with a 12 km grid resolution. The mother domain using a 36 km resolution covered most parts of China, Japan, Korea, Mongolia, and parts of South Asia to provide boundary conditions for domain 2. Based on the degree of contamination and haze frequency, we chose Shijiazhuang and Xingtai within domain 2 as the representative cities to analyze the haze pollution in the south Hebei [5].

The year 2007 was the most severe polluted year in the ten years from 2001 to 2010 [5, 6]. The simulation results of Shijiazhuang and Xingtai in July and December of 2007 by CMAQ were credible and would be suitable for further atmospheric research [7]. Considering the above reasons, we fixed on July and December of 2007 as the modeling period. 

### 2.2. Model Configuration and Input

The US Environmental Protection Agency (US EPA) Models-3/CMAQ modeling system version 4.7.1 with the process analysis tool was employed to explore the source of haze and PM_2.5_ in Shijiazhuang and Xingtai city. Two domains were sent up for CMAQ modeling system on Lambert projection: domain 1 was constituted of 164 × 97 grid cells on 36 km horizontal resolution, and 93 × 111 grid cells comprised domain 2 with 12 km horizontal resolution. The vertical resolution was stretched from the surface to the model top (100 mb), and 14 sigma layers were used for the vertical resolution of CMAQ with sparser layers at higher altitudes.

A clean atmospheric profile was used to provide the initial and boundary conditions required by the first day's simulation, while the subsequent data required was obtained from the CMAQ Chemistry-Transport Model (CCTM). For the purpose of eliminating the influence of error associated largely with the conditions, a spin-up period of five days was set.

MM5 model version 3.7 with four-dimensional data assimilation was conducted to provide CCTM with the meteorological parameters of the domain defined. There existed twenty-three sigma layers in the vertical grid structure. Two domains and one-way nesting were adopted by MM5 in which a 170 × 97 and 100 × 111 grid was chosen for the 36 and 12 km resolution, respectively. The centers of them were −18 km, −288 km, 1182 km, and 1044 km, respectively. Of the MM5 input data, the terrain and surface data were drawn from the U.S. Geological Survey database. First guess fields with 1° × 1° resolution, 6-hour interval, and the initial conditions were extracted from the U.S. Geological Survey database. The observation data used in the objective analysis were based on information from the National Center for Environmental Prediction (NCEP) Final (FNL) Operational Global Analysis datasets. The MM5 output files were postprocessed by the Meteorology-Chemistry Interface Processor (MCIP) on an hourly basis.

The emissions used here were extracted from Intercontinental Chemical Transport Experiment-Phase B (INTEX-B) emission inventory established by Zhang et al. [8] which was modified and updated on the basis of Transport and Chemical Evolution Over the Pacific (TRACE-P) presented by Streets et al. [9]. The inventory includes the emissions of sulfur dioxide (SO_2_), nitrogen oxides (NO_*x*_), carbon monoxide (CO), nonmethane volatile organic compounds (NMVOCs), PM_10_, PM_2.5_, black carbon (BC), and organic carbon (OC) for Asian countries in 2006. To satisfy the need of this study, we regridded this inventory form 1° × 1° resolution to 36 km and 12 km resolution using the gridding technique presented by Streets et al. [9] and Woo et al. [10].

### 2.3. Scenarios and Contribution Analysis

In this study, the pollution contribution from different emission sectors was analyzed. There were 26 modeling scenarios set up in this study, including one base case and twenty-five zero-emission scenarios of different areas and sectors. The following Table 1 explained the meanings of essential modeling scenarios for north China (domain 2) as we mentioned in this study.

Scenario analysis was conducted by cancelling the emissions of a sector and keeping emissions of other sectors to compare between the simulation results and base case. The difference denotes contribution value to the target sectors. And the ratio of contribution value to base case reflects contribution rate to pollutants concentration of target sectors. Hence, the following equation was used to estimate contribution rate:

(1) $$\begin{array}{c} C_{i,\text{contrib}.}=C_{\text{Base}}-C_{i-0},\\ P_{i,\text{contrib}.}=\frac{C_{i,\text{contrib}.}}{C_{\text{Base}}}, \end{array}$$

where *C*_*i*,contrib._ and *P*_*i*,contrib._ represent the pollution contributions of concentration and percentage, respectively, from sectors *i*. And *C*_Base_ and *C*_*i*−0_ mean the predicted concentrations of the base case and the case with zero emissions in sectors *i*, respectively. At present, several modeling studies have performed applying this methodology [5, 11–15] in north China.

## 3. Results and Discussions

### 3.1. Model Evaluation

**Figure 2:**
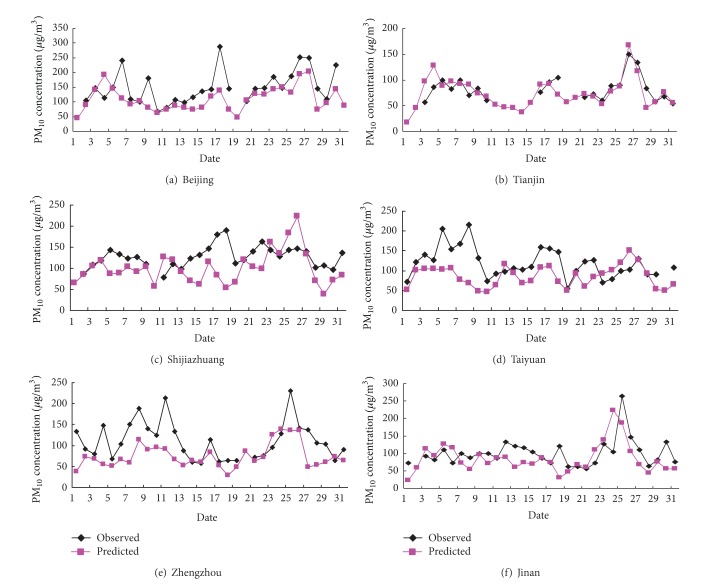
Comparison of observed and simulated daily average PM_10_ concentration in July 2007.

This study investigated the six representative cities in the simulated domain, Beijing, Tianjin, Shijiazhuang, Taiyuan, Zhengzhou, and Jinan, for the evaluation of the simulated results. To gain the limited air quality observation data for the model evaluation, we adopted the Air Pollution Index (API) data from Ministry of Environmental Protection of China (MEP) (http://datacenter.mep.gov.cn/), which reported daily value of most areas in China. According to the key pollutant API, the daily average concentration of each city could be back calculated [5, 11, 13]. The concentration of monitored special pollutants, such as PM_2.5_, PM_10_, SO_2_, and NO_*x*_, may not be available because only the API of the key pollutant was reported. The day with API less than 50 was considered to be a “clean day.” The key pollutant of the six representative cities was a coarse particulated matter (PM_10_) in most days of July and December 2007, so that this study only used PM_10_ concentration for model evaluation except for very few clean days and the days with SO_2_ as a key pollutant. The modeling results for December 2007 have been thoroughly evaluated in the study of Wang et al. [5], so only the results for July were showed in Figure 2.

From Figure 2, it can be seen that the observed and predicted values of PM_10_ daily concentration were overall in good agreement with each other, and the predicted data was lower than the observed ones in most of days and cities. The best consistence appears in Tianjin city. The average observed and predicted values of this city were 84 *μ*g·m^−3^ and 74 *μ*g·m^−3^, respectively, with the normalized mean bias (NMB) of only 12%. Followed by Jinan city, the average observed and simulated values of PM_10_ monthly concentration were 100 *μ*g·m^−3^ and 85 *μ*g·m^−3^, respectively, with NMB of 15%. The simulated mean monthly PM_10_ concentrations of the rest of cities were 100 *μ*g·m^−3^, 110 *μ*g·m^−3^, and 86 *μ*g·m^−3^, respectively, compared with the corresponding mean observed values of 127 *μ*g·m^−3^, 146 *μ*g·m^−3^, and 115 *μ*g·m^−3^, respectively. The NMBs of them were 21%, 25%, and 25%, respectively. Among those cities, Zhengzhou had relatively poor simulated performance, especially for the high PM_10_ concentration parts, NMB of which was up to 33%. However, the variation of simulated and observed values was consistent. Generally, the MM5-CMAQ modeling results for July and December in 2007 were credible so that it can be employed to analyze the air quality of these areas. 

### 3.2. Contributions by Local Sectors

The sectors analyzed in this study for the local (Hebei) sources include the power plant, the domestic combustion, the domestic noncombustion, and the industrial and traffic emissions.

**Figure 3:**
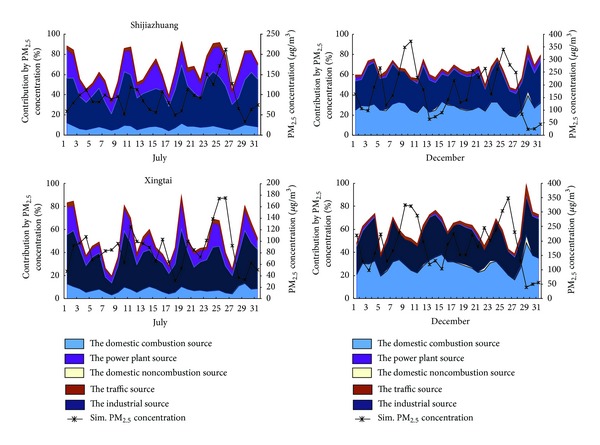
Contribution ratios of sector sources of Hebei area to daily average PM_2.5_ concentration in Shijiazhuang and Xingtai.

The contributions of different sectors from Hebei area to the PM_2.5_ concentrations in Shijiazhuang and Xingtai and the corresponding PM_2.5_ concentrations in July and December 2007 were summarized in Figure 3. In general, the average PM_2.5_ daily concentrations in December were higher than that in July, along with more appearing haze days, which might be related to the worse diffusion condition and the heating in December. In July 2007, the highest contribution ratios of mean daily PM_2.5_ concentration to Shijiazhuang were the industrial source and the power plant source, which contributed with an average ratio of 38.3% and 18.5%, respectively, followed by the domestic combustion source (7.7%) and the traffic source (2.7%). And the contribution ratio of the domestic noncombustion was almost zero. In December 2007, the largest contributor was still the industrial source, with average contribution ratio of 31.8%. At that time, the second largest contributor was the domestic combustion source (27.5%), higher than the power plant (2.5%) which was the second largest in July. This result might be due to the coal combustion for heating in winter. The traffic source and the domestic noncombustion source only provide 2.1% and 0.7% on average to the contribution ratio. Compared with Shijiazhuang in July 2007, the industrial source and the power plant source in Xingtai were still the main contributors, with average contribution ratios of 30.4% and 11.8%, respectively, followed by the domestic combustion source of 7.7%, the traffic source of 2.2%, and the domestic noncombustion source of zero. The largest contributor was the domestic combustion source with an average contribution of 29.1% which was higher than the industrial source with that of 28.3% in December 2007. The average contribution ratios of the traffic source domestic noncombustion source, and the power plant source were 2.3%, 0.9%, and 0.5%, respectively.

In summary, the industrial source was the major contributor to the average PM_2.5_ daily concentration in Shijiazhuang and Xingtai in July and December 2007. Furthermore, the domestic combustion source became the second contributor in December instead of the power plant source in July, which might be significantly related to the heating in winter across the Hebei areas. The power plant source held few contribution ratios in December compared with that in July, and the traffic source always kept a stable level in those two cities during the July and December 2007.

### 3.3. Contributions by Regional Sectors

**Figure 4:**
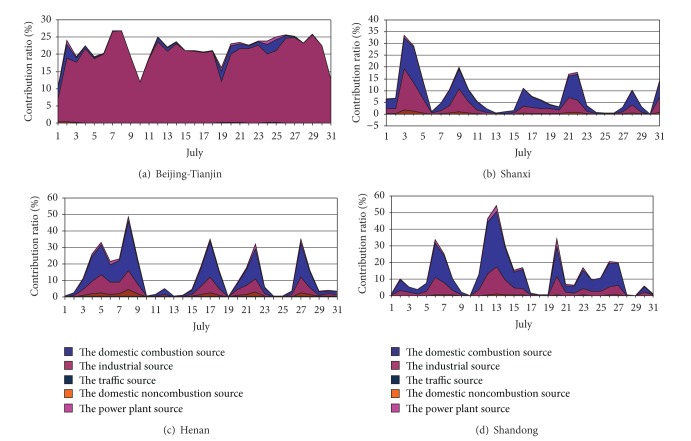
Contribution ratios of daily average PM_2.5_ concentration from sector sources of the surrounding areas to Shijiazhuang in July 2007.

**Figure 5:**
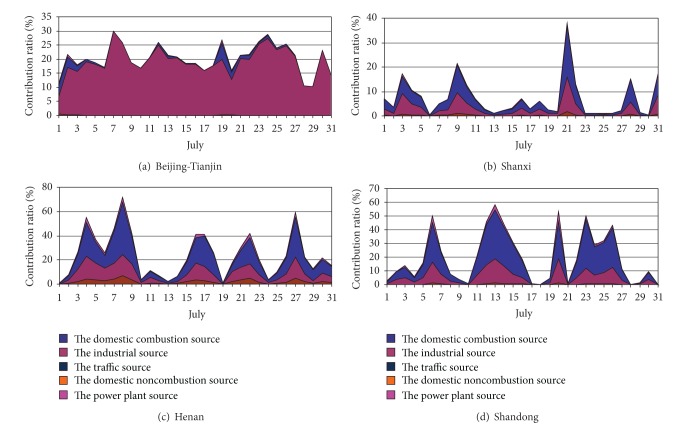
Contribution ratios of daily average PM_2.5_ concentration from sector sources of the surrounding areas to Xingtai in July 2007.

The contributions by the regional sectors to the average concentrations of PM_2.5_ at Shijiazhuang and Xingtai were summarized in Figures 4 and 5. As shown in Figures 4 and 5, the main regional contributors to Shijiazhuang and Xingtai were contributed by industrial source from Beijing-Tianjin; the mean contribution ratios were 20.3% and 19.1%, and the maximum contribution can be as high as 29.8% and 26.8%, respectively. In addition, the major contribution ratios were from the industrial source and the power plant source in Shanxi (3.2% and 4.3% for Shijiazhuang; 2.7% and 3.8% for Xingtai), Henan (3.7% and 7.5% for Shijiazhuang; 7.5% and 13.0% for Xingtai) and Shandong (3.6% and 5.2% for Shijiazhuang; 5.1% and 7.6% for Xingtai), in July. And the contribution ratio of Henan and Shandong was obviously larger than Shanxi. Another evident characteristic was that when the contribution ratios of the industrial source and the power plant source of Hebei decreased, the other areas increased. In particular, the variation of the contribution ratio of Henan and Shandong was completely conversed with Hebei. It indicated that the industrial source and the power plant source of Henan and Shandong became the main contributors when the contribution ratio of Hebei decreased in July. However, the contribution ratio of Shanxi did not present consistency as Henan and Shandong in July.

All in all, the industrial source and power plant source were the most evident contributors to PM_2.5_ in Shijiazhuang and Xingtai in July 2007, and the industrial source and power plant source were also the most important external contributors along with 21.2% to Shijiazhuang and 25.7% to Xingtai from the industrial source of the four external regions and 17.5% to Shijiazhuang and 24.9% to Xingtai from the power plant source of the four external regions.

**Figure 6:**
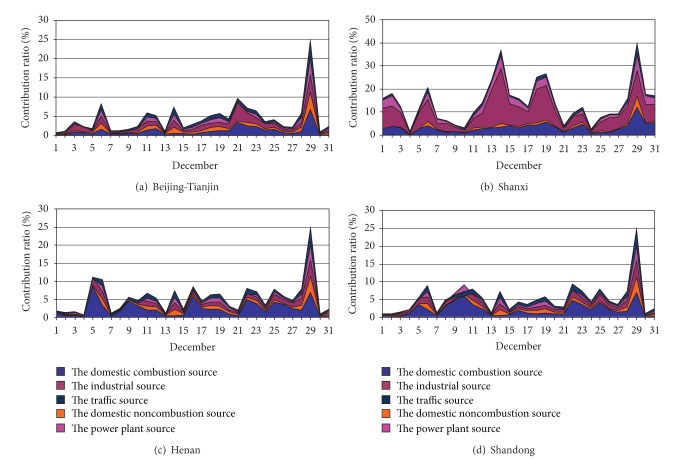
Contribution ratios of daily average PM_2.5_ concentration from sector sources of the surrounding areas to Shijiazhuang in December 2007.

**Figure 7:**
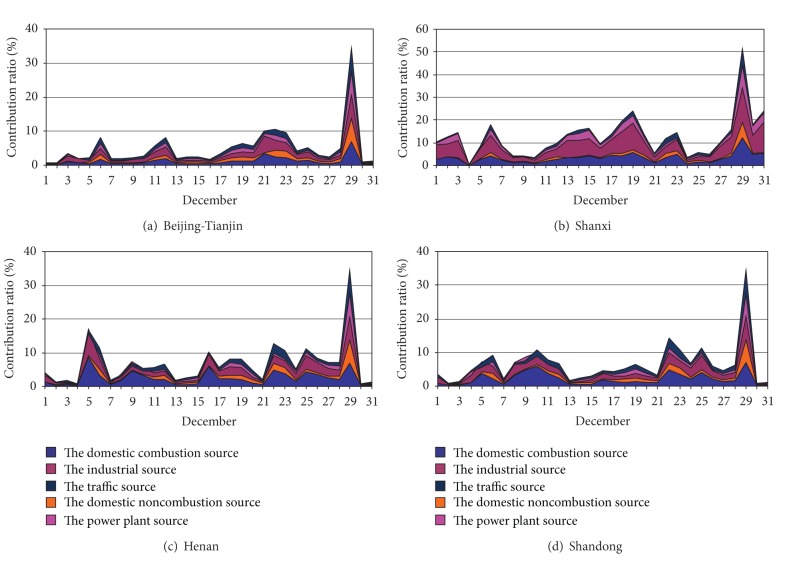
Contribution ratios of daily average PM_2.5_ concentration from sector sources of the surrounding areas to Xingtai in December 2007.

Comparison with contribution rate of July and December used by Figures 4–7, the contribution rate of the external sources decreased to some extent, especially the power plant source. The contribution of domestic combustion source, domestic noncombustion source, and transport source increased, especially for domestic combustion source and domestic noncombustion source. Domestic noncombustion source did not present in July, because its contribution was very little. The industrial source of Beijing-Tianjin was no longer the important source just as in July. But the industrial source and the domestic combustion source were the main contributors of external PM_2.5_. The contribution ratios of the domestic combustion source of Beijing-Tianjin, Shanxi, Henan, and Shandong to Shijiazhuang and Xingtai were 1.0% and 1.2%, 3.8% and 3.3%, 1.2% and 2.4%, and 1.3% and 2.1%, respectively. The contribution ratios of the industrial source of Beijing-Tianjin, Shanxi, Henan, and Shandong to Shijiazhuang and Xingtai were 1.2% and 1.4%, 7.0% and 5.5%, 1.0% and 1.9%, and 1.1 and 1.8%, respectively. The sources of the other areas provided a few PM_2.5_ at a low amount; Shanxi became the evident contributor of PM_2.5_ in the industrial source and the domestic combustion source.

In brief, the industrial source was also the main contributor to Shijiazhuang and Xingtai in December. The domestic combustion source exceeded the power plant source and became the second top highest source. In addition, the domestic noncombustions and the transport source both increased slightly. All the variation of the contribution rate could be correlation with the emission and synoptic condition of the different month, especially synoptic condition [15, 16].

## 4. Conclusions

This study estimated the sectoral contributions of the local the regional sources to the PM_2.5_ concentration over Shijiazhuang and Xingtai cities, which might be the most polluted cities over China. In summary, the industrial source and power plant source from the local area are the most evident contributors to PM_2.5_ in Shijiazhuang (38.3% and 18.5%) and Xingtai (30.4% and 11.8%) in July 2007, followed by the domestic combustion source (7.7% in both city) and the traffic source (2.7% in Shijiazhuang and 2.2% in Xingtai). In December 2007, the largest contributors were the industrial source in Shijiazhuang (31.8%) and the domestic combustion source in Xingtai (29.1%), followed by domestic combustion source in Shijiazhuang (27.5%) and industrial source in Xingtai (28.3%), then the power plant source (2.5%), the traffic source (2.1%), and the domestic noncombustion source (0.7%) in Shijiazhuang, and in Xingtai the traffic source, domestic noncombustion source, and the power plant source were 2.3%, 0.9%, and 0.5%, respectively. The power plant source, held few contribution ratios in December compared with that in July, and the traffic source always kept a stable level in the two cities during July and December 2007.

As to the regional sources, the industrial source was the major contributor for the average PM_2.5_ daily concentration no matter what in Shijiazhuang and Xingtai in July 2012, mainly from Beijing-Tianjin (20.3% and 19.1%), Shanxi (3.2% and 2.7%), and Henan (3.7% and 7.5%), followed by power plant and domestic combustion sources. The power plant source also was a nonignorable external contributor along with 17.5% to Shijiazhuang and 24.9% to Xingtai from the power plant source of the four external regions. In December, the major contributors were the domestic combustion source and the industrial source of the external regions; for example, Shanxi province was the biggest contributor in the domestic source and the industrial source in December, the contribution ratio of combustion source and the industrial source of Shanxi to Shijiazhuang was 3.8% and 7.0% and to Xingtai was 3.3% and 5.5%, respectively.

## Figures and Tables

**Table 1:** Modeling scenarios in this study.

Scenario name	Emission Scenario	Remarks of emission scenarios
Base	Base case	Baseline for contribution analysis
HB-POW0	Power plant emissions were turned off in Hebei province	To estimate the contribution of the power plants emissions from each area
BT-POW0	Power plant emissions were turned off in Beijing-Tianjin region	
HN-POW0	Power plant emissions were turned off in Henan province	
SD-POW0	Power plant emissions were turned off in Shandong province	
SX-POW0	Power plant emissions were turned off in Shanxi province	

HB-IND0	Industrial emissions in Hebei province were turned off	To estimate the contribution of industrial emissions from each area
BT-IND0	Industrial emissions were turned off in Beijing-Tianjin region	
HN-IND0	Industrial emissions were turned off in Henan province	
SD-IND0	Industrial emissions were turned off in Shandong province	
SX-IND0	Industrial emissions were turned off in Shanxi province	

HB-DOB0	Domestic combustion emissions were turned off in Hebei province	To estimate the contribution of domestic combustion emissions from each area
BT-DOB0	Domestic combustion emissions were turned off in Beijing-Tianjin region	
HN-DOB0	Domestic combustion emissions were turned off in Henan province	
SD-DOB0	Domestic combustion emissions were turned off in Shandong province	
SX-DOB0	Domestic combustion emissions were turned off in Shanxi province	

HB-DON0	Domestic noncombustion emissions were turned off in Hebei province	To estimate the contribution of domestic noncombustion emission from each area
BT-DON0	Domestic noncombustion emissions were turned off in Beijing-Tianjin region	
HN-DON0	Domestic noncombustion emissions were turned off in Henan province	
SD-DON0	Domestic noncombustion emissions were turned off in Shandong province	
SX-DON0	Domestic noncombustion emissions were turned off in Shanxi province	

HB-TRA0	Traffic emissions were turned off in Hebei province	To estimate the contribution of traffic emissions from each area
BT-TRA0	Traffic emissions were turned off in Beijing-Tianjin region	
HN-TRA0	Traffic emissions were turned off in Henan province	
SD-TRA0	Traffic emissions were turned off in Shandong province	
SX-TRA0	Traffic emissions were turned off in Shanxi province	
